# Pediatric Parapneumonic Empyema: Trends in the Post-COVID-19 Era in Northern India

**DOI:** 10.7759/cureus.104602

**Published:** 2026-03-03

**Authors:** Sujaya Mukhopadhyay, Brajendra Singh, Ruchika Bhatnagar, Rakesh Gupta, Rajeev Kumar, Sanju Yadav

**Affiliations:** 1 Pediatrics, Government Institute of Medical Sciences, Greater Noida, IND; 2 Pediatrics, Kalyan Singh Government Medical College, Bulandshahr, IND

**Keywords:** ambispective, intrapleural fibrinolytics, pediatric parapneumonic empyema, post covid-19 era, tb empyema

## Abstract

Introduction

The coronavirus disease 2019 (COVID-19) pandemic disrupted immunization services, and regular checkups were hampered, as the entire health infrastructure was focused on the management of the COVID-19 pandemic. During the pandemic, infectious diseases decreased because of a number of factors. This study was conducted to investigate changes in the incidence of empyema cases in the post-COVID-19 era, in comparison to pre-COVID-19 times.

Methods

This was an ambispective observational study of all cases of thoracic empyema admitted under the Department of Pediatrics in the Government Institute of Medical Sciences, Greater Noida, Uttar Pradesh, India, over a period of one and a half years, from June 2023 to December 2024, after the COVID-19 pandemic ended. The objectives were to evaluate the clinical characteristics, outcomes, and frequency of empyema cases after the COVID-19 pandemic. Patients aged 1 month to 14 years with pneumonia and empyema were included. Demographic and clinical parameters, presence of any comorbid illnesses, nutritional status, details of investigations, and other relevant information were collected in a predesigned case record form.

Results

The percentage of empyema cases during the study period was 0.56%. The majority of patients were below five years of age. Pneumonia was the most common predisposing factor. The percentage of tuberculosis empyema cases was also higher than in earlier periods; culture positivity was low, and fibrinolytics were used in a higher percentage of patients.

Conclusions

In this region, in post-COVID-19 times, the rate of pediatric parapneumonic empyema cases has decreased. The use of intrapleural fibrinolytic therapy (IFT) has increased, and surgical intervention can often be avoided if fibrinolytic therapy is used promptly.

## Introduction

The coronavirus disease 2019 (COVID-19) pandemic was declared to have ended on May 5, 2023, by the World Health Organization (WHO) [1]. The COVID-19 pandemic disrupted immunization services, and regular checkups were hampered, as the entire health infrastructure was focused on the management of the COVID-19 pandemic. During the pandemic, infectious diseases decreased because of a number of factors. Widespread lockdowns limiting the movement of people and transmission of microbes, continuous use of face masks, frequent handwashing, and use of personal protective equipment are some of the reasons. The pandemic had a negative impact on infections like scabies, and its incidence increased, probably because it forced people to live in close contact [2]. However, there are reports suggesting that tuberculosis also increased following the COVID-19 pandemic [3]. Pneumonia is a common cause of morbidity and mortality in children, especially those under five years of age [4]. Etiological agents vary with different age groups; they can be caused by organisms such as *Streptococcus pneumoniae*, *Staphylococcus aureus*, *Haemophilus influenzae* type B, respiratory viruses, *Mycobacterium tuberculosis*, and others. Common predisposing factors for pneumonia in the pediatric age group include incorrect feeding practices, such as non-exclusive breastfeeding, improper complementary feeds, undernutrition, anemia, air pollution, and lack of immunization [5]. Immunization, especially pneumococcal vaccination, has helped decrease the overall incidence of pneumonia in children. Empyema thoracis is a complication of pneumonia, and one-fourth of pediatric pneumonias are known to be complicated by syn-pneumonic effusion and empyema. It is also seen in pulmonary tuberculosis, though less frequently [6,7].

The study was conducted with the objective of evaluating the clinical characteristics, outcomes, and frequency of empyema cases after the COVID-19 pandemic ended.

## Materials and methods

This ambispective observational study is an analysis of all cases of thoracic empyema admitted under the Department of Pediatrics in the Government Institute of Medical Sciences, Greater Noida, Uttar Pradesh, India, over a period of one and a half years - from June 2023 to December 2024 - after the COVID-19 pandemic ended. The study was approved by the Institute’s Ethics Committee (GIMS/IEC/HR/2024/58). 

Patients aged 1 month to 14 years with pneumonia, a high white blood cell count, an elevated serum level of C-reactive protein, and pleural effusions fulfilling at least one of the following criteria were included in this study: (i) frank pus or purulent-appearing fluid on diagnostic pleural aspiration, (ii) positive pleural fluid culture, and (iii) positive pleural fluid Gram stain. Empyema secondary to diagnostic or therapeutic thoracentesis, pleural biopsy, or tube thoracostomy for spontaneous pneumothorax or malignant pleural effusion was not included.

**Figure 1:**
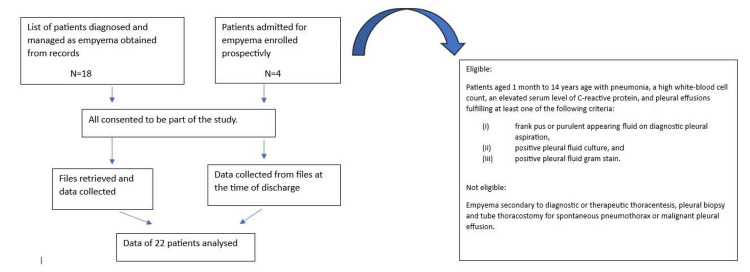
Flow of study

Informed consent was taken telephonically from those already discharged, and case record forms were filled out after retrieving data from the hospital records. For patients who were currently hospitalized, informed consent was taken, and data were collected from the records at the time of discharge to obtain complete information (Figure 1).

The following data were collected in a predesigned case record form: the demographic and clinical parameters, including age and sex; symptoms (fever, cough, chest pain, dyspnea, expectoration, and hemoptysis) and their duration; presence of any comorbid illness; nutritional status as per the WHO criteria; immunization status, including COVID-19 vaccination (children who were not fully immunized for age, as per national guidelines, were classified as partially immunized); chest radiographs; ultrasound (USG) and computed tomography (CT) chest reports [8]; and reports of thoracocentesis, including cytology, biochemistry, culture, and sensitivity. Complete blood counts (CBCs), renal function tests (RFTs), liver function tests (LFTs), blood culture, and Cartridge-Based Nucleic Acid Amplification Test (CBNAAT) reports, if done, as well as treatment taken and use of intrapleural fibrinolytics, were documented. Time to fever resolution, duration of pleural drainage, duration of hospitalization, and need for surgery were recorded to assess progress and outcomes.

Data were analyzed using IBM SPSS Statistics for Windows, Version 28 (Released 2021; IBM Corp., Armonk, NY, USA). Normally distributed data are presented as means ± standard deviation (SD), while non-normally distributed data are expressed as medians with interquartile ranges (IQRs). Categorical data, such as sex, clinical symptoms, immunization status, and radiological findings (chest X-ray, USG, and CT), are reported as absolute frequencies and percentages. Clinical outcomes, including time to fever resolution and duration of hospitalization, were summarized using descriptive statistics to evaluate patients’ progress.

## Results

The total number of patients aged 1 month to 14 years, admitted during the study period in the pediatric department, was 3928. Out of these, 22 were diagnosed with empyema, representing 0.56% of total pediatric admissions. The majority - nine (40.9%) children - were in the age group of 1-5 years, followed by six (27.3%) children in the 5-10 years group. The male-to-female ratio was 0.83, with more female patients. Fifteen (68.2%) children were fully immunized according to age, seven (31.8%) were incompletely immunized, and two (9.1%) were unimmunized. In terms of nutritional status, eight (36.3%) patients were underweight, and four (18.2%) were wasted. The most common presentation was fever in all patients, 22 (100%), followed by cough in 21 (95.5%) and breathlessness in 18 (81.8%). Five (22.7%) patients had abdominal pain with fever as a presenting feature (Table 1). The majority - 45.5% of patients - had fever for 7-14 days, and 40.9% had a cough of 7-14 days’ duration (Table 2).

**Table 1:** Clinical and demographic characteristics of the study patients CT chest: Computed tomography of the chest

Characteristics		Number of cases N (%), n = 22
Clinical features	Fever	22 (100)
	Chest pain	12 (54.5)
	Cough	21 (95.5)
	Breathlessness	18 (81.8)
	Pain abdomen	5 (22.7)
Age groups (years)	0-1	3 (13.6)
	1-5	9 (40.9)
	5-10	6 (27.3)
	>10	4 (18.2)
Gender	Male	10 (45.5)
	Female	12 (54.5)
Immunization status	Complete	15 (68.2)
	Incomplete	7 (31.8)
	Unimmunized	2 (9.1)
Nutritional status	Normal	10 (45.5)
	Underweight	8 (36.3)
	Wasted	4 (18.2)
Investigations	CT chest showing loculations/septations	15 (68.2)
	Blood culture positivity	2 (9.1)
	Pleural fluid culture positivity	3 (13.6)
Etiology of empyema	Post pneumonic	19 (86.4)
	Tubercular	3 (13.6)

**Table 2:** Average duration of symptoms

Duration (days)	Fever N (%)	Cough N (%)	Difficulty breathing N (%)	Chest pain N (%)
<7	4 (18.2)	7 (31.8)	16 (72.7)	18 (81.8)
7-14	10 (45.5)	9 (40.9)	6 (27.3)	4 (18.2)
>14	8 (36.4)	6 (27.3)	0	0

On evaluation, CT chest showed loculations and/or septations in 15 (68.2%) cases. One (4.5%) patient was also found to have a hepatic abscess. Culture positivity was low. In three (13.6%) patients, pleural fluid culture was positive, while blood culture was positive in two (9.1%) cases. Out of a total of five positive culture reports (three pleural fluid and two blood), *S. aureus* was identified in three patients and *S. pneumoniae* in two patients (Table 1). In 19 (86.4%) patients with empyema, the etiology was pneumonia, while in three (13.6%) patients, it was tubercular in origin (Table 1).

All patients were treated with antibiotics, and antitubercular treatment (ATT) was started in three (13.6%) patients after evaluation. Fibrinolytics were used in 15 (68.2%) patients. One (4.5%) patient required decortication (Table 3). In terms of complications, collapse was the most common complication, seen in four (18.2%) cases (Table 3). The median time to become afebrile was five days (IQR: 10-22), and the median duration of hospital stay was 17 days (IQR: 13.5-25). All 22 (100%) patients were discharged successfully (Table 4).

**Table 3:** Treatment given and complications ATT, antitubercular treatment

Treatment given	Number (%)
Antibiotics	22 (100)
ATT	3 (13.6)
Intra-pleural fibrinolytics	15 (68.2)
Decortication	1 (4.5)
Complications	Number (%)
Air leaks	3 (13.6)
Bronchopleural fistula	2 (9.1)
Collapse	4 (18.2)
Pleural thickening	1 (4.5)

**Table 4:** Distribution of outcome parameters ICTD, intercostal tube drainage; IQR, interquartile range

Outcome parameters	Median (IQR)
Time taken to become afebrile	5 (10-22)
Duration of ICTD insertion	14.50 (3-6.25)
Duration of hospital stay	17 (13.5-25)
Discharge N (%)	22 (100)
Expiry N (%)	0

## Discussion

In this ambispective study from a tertiary care hospital, we found that, in this region, in post-COVID-19 times, the rate of empyema cases has decreased, and most of the affected children were below five years of age [6]. Pneumonia was the most common predisposing factor, although the percentage of tuberculosis empyema cases was higher than in earlier periods. Culture positivity was low, and fibrinolytics were used in a higher percentage of patients.

The percentage of empyema cases in our institute was 0.56%, which is lower than that reported in other studies from India. In a study by Rao and Chandra, 1.44% of cases admitted to the pediatrics department were empyema [9]. Similarly, in 2016, Ramireddy et al. reported that empyema cases comprised 1.26% of the total admissions [10]. This trend was also reported internationally in a study by Chan et al. [11]. They compared the incidence, etiology, and outcomes of patients admitted for pleural empyema in Hong Kong during the pre-COVID-19 (January 2015-December 2019) and post-COVID-19 (January 2020-June 2022) periods and found a marked decline in the incidence of pleural empyema in children (pre-COVID-19, 18.4 ± 4.8 vs. post-COVID-19, 2.0 ± 2.9 cases per year; p = 0.036).

This decrease in empyema cases may be due to the tendency of people to seek consultation and start antibiotics earlier, as antibiotic use has increased tremendously worldwide after the COVID-19 pandemic. This is evident from several national and international studies, such as that by Mandal et al. [12]. They highlighted that the antibiotic prescription rate was higher in the private sector and that newer antibiotics are commonly used. The prevalence of antibiotic use was 36.77% in their study. A multinational study across six European countries by Kostev et al. [13] found that, in the post-COVID-19 period, antibiotic prescriptions in outpatient clinics have increased. A similar trend was also seen in Singapore [14]. The higher and more prompt use of antibiotics could be one reason why fewer patients with pneumonia progressed to empyema. However, this trend in antibiotic use is concerning and may lead to antibiotic resistance.

Fever, cough, and chest pain were the most common presenting complaints. In 45.45% of patients, fever had been present for 7-14 days, and in 40.91%, cough had persisted for 7-14 days before presentation to a health facility. It was observed that the onset of chest pain and difficulty breathing prompted patients to report to the hospital earlier. Other studies have reported similar symptoms, with a usual duration of more than seven days [15,16]. Abdominal pain was an unusual symptom, seen in 22.7% of patients, and was localized to the upper abdomen. No abdominal pathology was detected in 9.1% of patients, while 4.5% of patients had a liver abscess. The pain in those without any abdominal pathology was probably referred pain. In a study by Sharma et al., abdominal pain was seen in 13.3% of cases [6].

In this study, 54.5% of patients were below five years of age, with infants comprising 13.6% of the total. A similar age group was found to be affected in other studies from India [6,17]. Pneumonia and its complications are more frequent when there are predisposing conditions affecting a child’s immune status, such as malnutrition, HIV, overcrowding at home, and parental smoking. A history of measles infection in the previous three months was present in 9.1% of cases; 54.5% had various degrees of malnutrition, and 31.8% of cases were partially immunized or unimmunized.

Children are susceptible to bacterial infections, causing pneumonia and, consequently, empyema. *H. influenzae* type B, *S. pneumoniae*, and *S. aureus* are the most frequently isolated microorganisms. *S. aureus* is the dominant pathogen in developing countries, as reported in previous studies [15,18]. With the introduction of vaccination against *H. influenzae* type B and *S. pneumoniae* in the national immunization schedule, their incidence has decreased. In this study, although blood culture positivity was low, *S. aureus* was the main isolate. The rampant use of antibiotics before hospitalization could be a reason for the low culture positivity.

In 86.4% of cases, pneumonia was the cause of empyema, and in 13.6% of cases, tuberculosis was identified as the cause. As per previous reports, frank tuberculous empyema used to be uncommon, but recent studies report rates ranging from 2% to 13% [10,19,20]. We found a comparable rate of tubercular empyema in our study. One patient (4.5%) in this study had a hepatic abscess along with empyema. It was non-tubercular. Coexisting hepatic abscess and empyema were reported in 2.4% of cases by Sadani and Das in their study [21].

Treatment of empyema requires antibiotics and drainage of pus. Intercostal tube drainage (ICTD) was used in all patients. Fibrinolytic use has gained momentum, with intrapleural fibrinolytic therapy (IFT) reported to be comparable to video-assisted thoracoscopic surgery (VATS) [22]. We used chest drainage with fibrinolysis in those with septations or loculations on thoracic CT in 68.2% of patients.

In terms of outcome parameters, the median duration of hospitalization was 17 days (IQR: 27), and the time taken to become afebrile was five days (IQR: 12). All patients were successfully discharged.

Limitations

Not having data regarding pediatric parapneumonic empyema in the pre-COVID-19 pandemic from the same institute is one drawback of the study. We tried to overcome this by comparing it with data from the same region.

## Conclusions

We found that in this region, in post-COVID-19 times, the rate of pediatric parapneumonic empyema cases has decreased. Use of IFT has increased, and surgical intervention can be avoided if fibrinolytic therapy is used promptly. Future research could focus on antibiotic usage in this region, especially among patients admitted with serious infections. Additionally, studies are needed to identify the reasons for the increase in tubercular empyema cases in children post-COVID-19 pandemic.
